# Bisphenol S impairs courtship, grooming, and locomotor behaviors in *Drosophila melanogaster*

**DOI:** 10.17912/micropub.biology.001761

**Published:** 2025-10-24

**Authors:** Zubair Mahyar, Connor Spann, Chloe J. Welch, Judith L. A. Fishburn, Ricardo Cantua, Nivene Hojeij, Kevin Nzenkue, Adam Alfareh, Alexandra Davis, Mikayla Lerandeau, Kimberly Mulligan

**Affiliations:** 1 Biological Sciences, California State University, Sacramento, Sacramento, California, United States; 2 School of Biological Sciences, University of California, San Diego, San Diego, California, United States

## Abstract

Bisphenol S (BPS), a common substitute for bisphenol A (BPA) in "BPA-free" products, is a potential developmental neurotoxicant. To investigate the developmental consequences of BPS exposure, we used *Drosophila melanogaster* as a model organism. Following exposure to BPS, we assessed three distinct behaviors: adult courtship, adult grooming, and larval locomotion. BPS exposure resulted in attenuated courtship activity, impaired grooming behavior, and some instances of altered larval locomotion. These findings support the hypothesis that developmental exposure to BPS, similar to BPA and structurally related analogs, can disrupt behavioral outcomes.

**Figure 1. Developmental exposure to bisphenol S (BPS) affects behavioral phenotypes in Drosophila:**
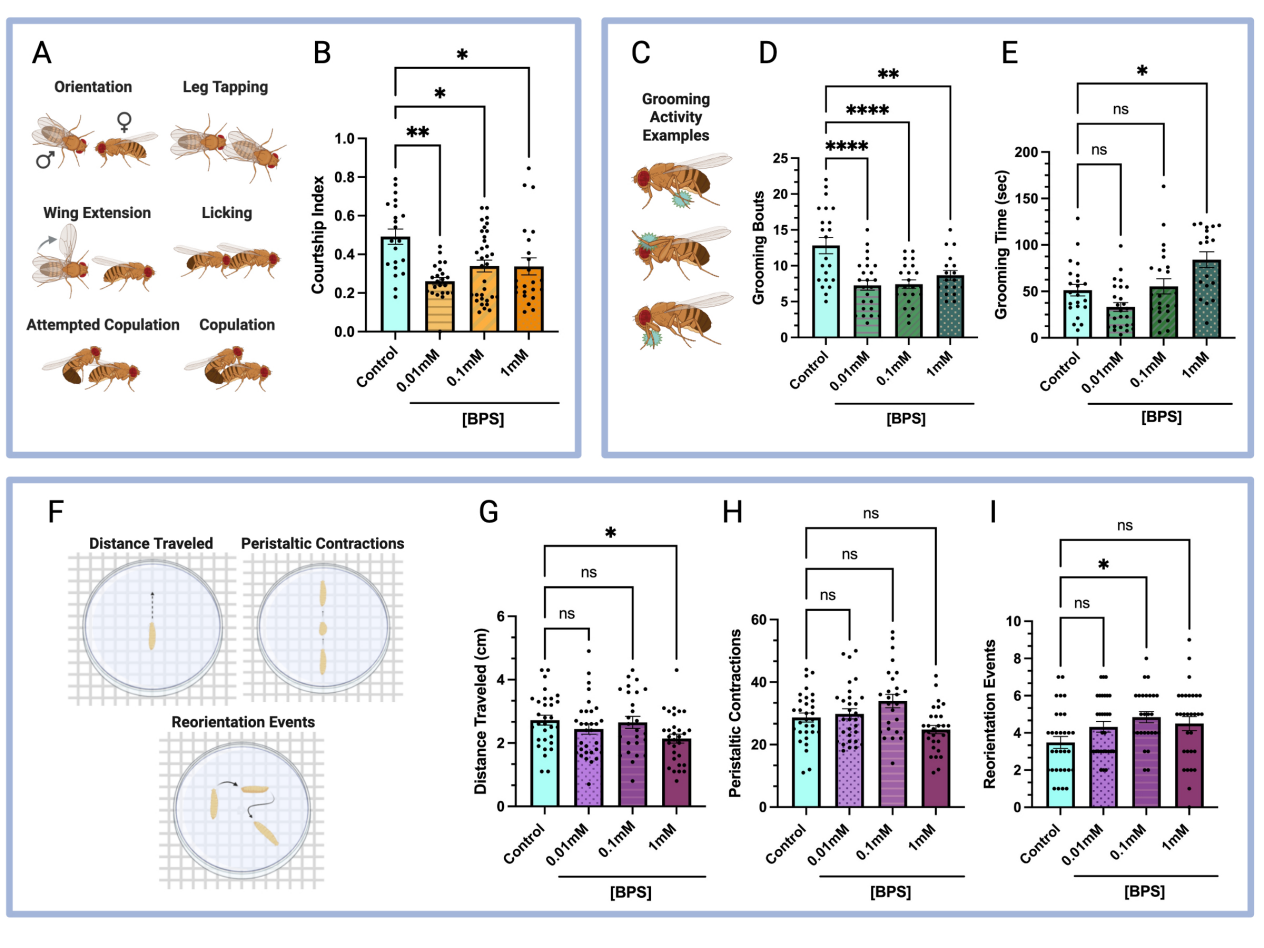
**(A)** Standard male courtship behaviors, including orientation/following, leg tapping, wing extension, genital licking, attempted copulation and successful copulation, were quantified. The courtship index was calculated by dividing the time spent participating in these behaviors by the total duration of the assay. **(B)** Courtship index: exposure to 0.01 mM, 0.1 mM, and 1 mM BPS significantly reduced the courtship index of flies compared to the unexposed control group. Sample sizes: unexposed, n = 21; 0.01 mM, n = 23; 0.1 mM, n = 33; 1 mM, n = 23. **(C)** Examples of repetitive adult grooming behaviors, including rubbing of the prothoracic or metathoracic legs against each other or other body parts. **(D)** Grooming bouts: exposure to 0.01 mM, 0.1 mM, and 1 mM BPS significantly reduced the number of grooming bouts performed by flies when compared to the unexposed control group. Sample sizes: unexposed, n = 22; 0.01 mM, n = 26; 0.1 mM, n = 23; 1 mM, n = 18. **(E)** Grooming time: exposure to 1 mM BPS significantly increased the time spent grooming, while 0.01 mM and 0.1 mM BPS had no significant impact. Sample sizes: unexposed, n = 22; 0.01 mM, n = 26; 0.1 mM, n = 23; 1 mM, n = 18. **(F)** Larval locomotor behaviors examined include total distance traveled, number of peristaltic contractions, and total reorientation events. **(G)** Distance traveled: exposure to 1 mM BPS significantly reduced the total distance traveled by larvae, while 0.01 mM and 0.1 mM BPS had no significant impact. Sample sizes: unexposed, n = 31; 0.01 mM, n = 31; 0.1 mM, n = 26; 1 mM, n = 30. **(H)** Peristaltic contractions: exposure to 0.01 mM, 0.1 mM, and 1 mM BPS had no significant impact on the number of contractions. Sample sizes: unexposed, n = 31; 0.01 mM, n = 31; 0.1 mM, n = 26; 1 mM, n = 30. **(I)** Reorientation events: exposure to 0.1 mM BPS significantly increased the number of reorientation events, while 0.01 mM and 1 mM BPS had no significant impact. Sample sizes: unexposed, n = 31; 0.01 mM, n = 31; 0.1 mM, n = 26; 1 mM, n = 30. (For all bar graphs, bars represent mean ± SEM; ns = not significant; * = P < 0.05; ** = P < 0.01; *** = P < 0.0001.)

## Description

Bisphenol S (BPS) is a chemical compound widely used in the production of polyethersulfone (PES) plastics, which are often marketed as a safer alternative to bisphenol A (BPA)-based polycarbonate materials (Lehmler et al. 2018). BPS-derived PES is found in a variety of rigid plastic products—for example, baby bottles, food packaging, and medical devices—as well as in synthetic fibers in textiles and clothing. Beyond plastics, BPS is also used as a color developer in thermal papers, like receipts, labels, and tickets. Products containing BPS are frequently marketed as “BPA-free,” a term consumers associate with reduced health risks (Scherer et al. 2014). However, growing evidence indicates that BPA analogs, including BPS, may pose similar threats to human health (Cantua and Mulligan 2025, Siracusa et al. 2018). Like BPA, BPS is a lipophilic compound capable of crossing cellular membranes (Villalain 2024) that can function as an endocrine-disrupting chemical (Rochester and Bolden 2015, Stanojevic and Sollner Dolenc 2025). Thus, a comprehensive assessment of BPS’s toxicological effects is needed, particularly given its increasing environmental prevalence and human exposure (Wu et al. 2025).

To advance our understanding of BPS, we examined behavioral outcomes in *Drosophila melanogaster* following developmental exposure, as a means of assessing potential neurodevelopmental toxicity. Our previous work has shown that BPA and bisphenol F (BPF), another common BPA analog, disrupt neurodevelopment in *Drosophila* , leading to altered gene expression, axon guidance defects, and behavioral impairments (Fishburn et al. 2024, Nguyen et al. 2021, Welch et al. 2022). Although limited, emerging research suggests that BPS may also interfere with neurodevelopment (Cantua and Mulligan 2025; Musachio et al. 2024; Santos Musachio et al. 2023). To evaluate this, we exposed *Drosophila* to BPS during development and assessed its impact on courtship behavior, grooming activity, and locomotion.

The courtship assay examines a series of genetically programmed, sequential behaviors performed by male flies ( Figure 1A ). Because this behavioral sequence relies on the integration of multiple sensory modalities, it serves as a sensitive indicator of potential neurodevelopmental impairments. To assess the impact of BPS on courtship activity, we used the courtship assay to measure the courtship index (CI), defined as the proportion of time a male engages in courtship behaviors during the assay period. Consistent with previous findings that BPA and BPF reduce the CI in *w ^1118^* flies (Fishburn et al. 2024, Nguyen et al. 2021), we found that BPS significantly decreased the CI of *w ^1118^* flies at all concentrations tested ( Figure 1B ). Specifically, CI values were significantly lower in flies exposed to 0.01 mM BPS (0.26 ± 0.09; *P* = 0.001), 0.1 mM BPS (0.34 ± 0.18; *P* = 0.017), and 1 mM BPS (0.34 ± 0.21; *P* = 0.022), compared to unexposed controls (0.49 ± 0.18).

*Drosophila* grooming behavior involves cleaning activities directed toward the body surface, and because it is dependent on the integration of multiple neural circuits, can act as an indication of potential disruptions in the neurodevelopmental program ( Figure 1C ) (Barradale et al. 2017). Grooming data can be represented by analyzing numbers of grooming bouts and total time spent engaging in the behavior. Previous findings demonstrated that exposure to both BPA and BPF affects grooming behaviors in adult male flies (Fishburn et al. 2024, Nguyen et al. 2021). We found that the number of grooming bouts performed by *w ^1118^* flies exposed to 0.01 mM BPS (7.30 ± 3.42; *P* < 0.0001), 0.1 mM BPS (7.43 ± 2.83; *P* < 0.0001), and 1mM BPS (8.67 ± 2.87; *P* = 0.002) were significantly reduced compared to unexposed controls (12.82 ± 5.32; Figure 1D ). Additionally, exposure to 1 mM BPS (84.1 sec ± 36.3; *P* = 0.044) significantly increased the overall time spent grooming compared to unexposed flies (51.3 sec ± 29.3), whereas 0.01 mM and 0.1 mM BPS had no significant effect ( Figure 1E ).

Fruit fly larvae exhibit crawling behavior as they forage for food and respond to environmental stimuli (Clark et al. 2018). This form of navigation involves locomotion driven by anterior-to-posterior peristaltic contractions, interspersed with pauses and directional changes (Lahiri et al. 2011). Previous studies have shown that exposure to BPA and BPF significantly alters *w ^1118^* larval locomotion (Fishburn et al. 2024, Nguyen et al. 2021). Here, we assessed the impacts of BPS on the total distance traveled, number of peristaltic contractions, and the number of reorientation events performed by late third instar larvae ( Figure 1F ). Exposure to 1 mM BPS significantly reduced the total distance traveled by larvae (2.14 cm ± 0.790; *P* = 0.030) compared to the unexposed control group (2.72 cm ± 0.851), while 0.01 mM and 0.1 mM BPS had no significant effect ( Figure 1G ). Exposure to all three concentrations of BPS did not produce significant changes in the number of peristaltic contractions compared to control larvae ( Figure 1H ). However, we found a significant increase in the number of reorientation events upon exposure to 0.1 mM BPS (4.85 ± 1.51; *P* = 0.0132) compared to the control group (3.48 ± 1.77), but no significant change in the 0.01 mM or 1 mM BPS exposed larvae ( Figure 1I ).

In summary, exposure to BPS altered *Drosophila* courtship, grooming, and larval locomotor behaviors. BPS reduced courtship activity, a behavior that relies on the integration of sensory inputs and motor outputs through a number of specialized neural circuits (Pavlou and Goodwin 2013); therefore, its impairment potentially suggests dysfunction in sensory processing systems, neuromodulatory pathways, and/or motor control networks. BPS increased the total time spent grooming in a dose-dependent manner while reducing the total number of grooming bouts, again indicating a possible impact on neural and/or motor regulation. This specific grooming phenotype could reflect reduced grooming initiation coupled with prolonged bouts, potentially caused by behavioral rigidity, or it could indicate impaired behavioral sequencing, in which flies become fixated on a specific grooming task and struggle to transition between movements (Seeds et al. 2014). BPS affected larval locomotor activity in a selective, dose-dependent manner; at the highest concentration tested (1 mM), larvae exhibited a significant reduction in total distance traveled, suggesting diminished locomotor output potentially due to effects on neural circuits governing movement or motivation. However, the number of peristaltic contractions remained unchanged across all concentrations, indicating that BPS does not impair basic motor function or muscle activity. Interestingly, exposure to 0.1 mM BPS significantly increased the number of reorientation events, revealing an alteration in navigational behavior or decision-making. Consistent with previous analyses of BPA and BPF on larval locomotion (Fishburn et al. 2024, Nguyen et al. 2021), this effect exhibited a non-linear pattern, as it was absent at both lower (0.01 mM) and higher (1 mM) concentrations. Notably, while larval peristalsis is primarily a reflexive motor behavior, reorientation involves more complex sensorimotor integration—like courtship and grooming behaviors in adults. This distinction in phenotypes suggests that developmental exposure to BPS primarily disrupts higher-order neural functions rather than basic motor circuits. Importantly, because we did not examine the underlying neural or molecular mechanisms, additional experiments are required to establish whether the observed behavioral effects reflect neurodevelopmental disruption or other physiological consequences of BPS exposure. Our results are consistent with a growing body of evidence that developmental exposure to BPS can impair behavior and may not be a safe alternative to BPA.

## Methods

*Fly husbandry*

*Drosophila* of the *w ^1118^* strain (Bloomington Stock #5905) were reared in a humidified incubator on a 12:12 hour light:dark cycle on cornmeal-yeast-agar based fly food (Bloomington Stock Center recipe).

*Bisphenol S (BPS) exposure*

BPS was dissolved in filtered water and incorporated into standard cornmeal-yeast-agar fly food, following the Bloomington Stock Center recipe. Three concentrations of BPS were used—1 mM, 0.1 mM, and 0.01 mM—alongside a water-only negative control. Virgin female flies were exposed to BPS-containing food for four days before the introduction of young males, ensuring oocyte (and thus embryonic) exposure. BPS exposure continued throughout F1 larval development, and all behavioral assays were conducted on the F1 generation at the larval or adult stage.

*Courtship*

Courtship behavior was assessed using a modified version of a previously established protocol (Markow and Hanson 1981). F1 naïve males were collected immediately after eclosion and housed individually in food vials under a 12:12 hour light:dark cycle for 5–7 days. Each male was then introduced into a standard courtship chamber containing an unexposed virgin female (5–7 days old). Courtship interactions were recorded for 10 minutes. Behaviors—including following/orientation, leg tapping, wing extension, licking, copulation attempts, and successful copulation—were scored by observers blinded to treatment conditions. The courtship index (CI) was defined as the total time a male engaged in any courtship behavior, divided by the 10-minute observation period.

*Grooming Activity*

The grooming assay was adapted from a previously established protocol (Sachs 1988). Male flies were collected and aged for four to six days in separate food chambers in a standard incubator before being transferred to an observation chamber via aspiration. Following a one-minute acclimation period, the flies were recorded for five minutes. Video recordings were analyzed by individuals blinded to exposure conditions, who quantified the percentage of time each fly spent grooming.

*Larval Locomotor Activity*

The larval locomotion assays were adapted from two established protocols (Kashima et al. 2017). Individual, age-matched late third instar larvae were placed in a 15-cm petri dish containing a 2% agarose gel. After a one-minute acclimation period, the distance traveled, number of peristaltic contractions, and orientation changes were recorded over a one-minute interval. Locomotor behaviors were scored by individuals blinded to exposure conditions.
